# Does Exposure to a Radiofrequency Electromagnetic Field Modify Thermal Preference in Juvenile Rats?

**DOI:** 10.1371/journal.pone.0099007

**Published:** 2014-06-06

**Authors:** Amandine Pelletier, Stéphane Delanaud, René de Seze, Véronique Bach, Jean-Pierre Libert, Nathalie Loos

**Affiliations:** 1 PériTox Laboratory, UMR-I 01 INERIS, Faculty of Medicine, Jules Verne University of Picardy, Amiens, France; 2 PériTox Laboratory, UMR-I 01 INERIS, Experimental Toxicology Unit, National Institute of Industrial Environment and Risks (INERIS), Verneuil-en-Halatte, France; University of Oxford, United Kingdom

## Abstract

Some studies have shown that people living near a mobile phone base station may report sleep disturbances and discomfort. Using a rat model, we have previously shown that chronic exposure to a low-intensity radiofrequency electromagnetic field (RF-EMF) was associated with paradoxical sleep (PS) fragmentation and greater vasomotor tone in the tail. Here, we sought to establish whether sleep disturbances might result from the disturbance of thermoregulatory processes by a RF-EMF. We recorded thermal preference and sleep stage distribution in 18 young male Wistar rats. Nine animals were exposed to a low-intensity RF-EMF (900 MHz, 1 V.m^−1^) for five weeks and nine served as non-exposed controls. Thermal preference was assessed in an experimental chamber comprising three interconnected compartments, in which the air temperatures (T_a_) were set to 24°C, 28°C and 31°C. Sleep and tail skin temperature were also recorded. Our results indicated that relative to control group, exposure to RF-EMF at 31°C was associated with a significantly lower tail skin temperature (−1.6°C) which confirmed previous data. During the light period, the exposed group preferred to sleep at T_a_ = 31°C and the controls preferred T_a_ = 28°C. The mean sleep duration in exposed group was significantly greater (by 15.5%) than in control group (due in turn to a significantly greater amount of slow wave sleep (SWS, +14.6%). Similarly, frequency of SWS was greater in exposed group (by 4.9 episodes.h^−1^). The PS did not differ significantly between the two groups. During the dark period, there were no significant intergroup differences. We conclude that RF-EMF exposure induced a shift in thermal preference towards higher temperatures. The shift in preferred temperature might result from a cold thermal sensation. The change in sleep stage distribution may involve signals from thermoreceptors in the skin. Modulation of SWS may be a protective adaptation in response to RF-EMF exposure.

## Introduction

People living near mobile phone base stations may be concerned about the health effects of the associated radiofrequency electromagnetic fields (RF-EMFs). Although a number of studies have found a relationship between the proximity of a base station antenna and the presence of symptoms (such as headache, memory changes, anxiety, sleep disturbance or feelings of discomfort) but this topic remains subject to debate [1], [2], [3]. In previous research, we found that rats exposed to low-intensity RF-EMF (900 MHz, 1 V.m^−1^) at an ambient temperature (T_a)_ of 31°C exhibited (i) unusually high peripheral vasoconstrictor tone and (ii) fragmentation of their paradoxical sleep (relative to non-exposed controls) [4]. The core temperature inside the brain was normal. Few experiments have been performed with low-intensity RF-EMFs (which do not produce a significant increase in central temperature through a direct heating effect) and it is still not clear whether and how behavioral thermal responses are elicited in this context. However, one can hypothesize that sleep disturbances are due to thermal discomfort induced by RF-EMFs. Previous studies of male adult squirrel monkeys exposed to high-intensity 2450 MHz RF-EMF (specific absorption rate (SAR): 1.1 to 3.2 W.kg^−1^) found that thermal comfort could be modified by exposure. Thus, the thermal preference was 2–3°C lower than normal as a result of exposure [5]. This change was associated with a higher hypothalamic temperature (0.2–0.3°C greater) [6] and was dependent on the intensity (but not the duration) of exposure [7]. In Adair et al.’s experiments, the behavioral thermoregulatory response might have been mediated centrally (rather than peripherally), since the hypothalamic temperature increased and the skin and rectal temperatures did not change.

On the basis of these literature reports, we hypothesized that the low skin temperature (due to high vasoconstrictor tone) might be accompanied by a change in thermal preference, since skin sensory afferents are strongly involved in the thermoregulatory behavioral response [8]. This response illustrates the anticipatory function of thermoregulatory behavior and enables the animal to avoid or rapidly escape from disturbances of the external environment. In the light of the observations reported above, one can legitimately hypothesize that the thermal comfort of RF-EMF-exposed animals might be shifted towards higher ambient temperatures. To the best of our knowledge, the putative relationship between thermal comfort and chronic exposure to low-intensity RF-EMF has not previously been investigated.

The air temperature selected by the animal is known as the thermal comfort temperature or preferred temperature [9], [10]. It is well known that the rat is able to detect small changes of T_a_, the direction of the thermal gradient and the location of the heat or cold source. With a viewing to probing the impact of a putative change in peripheral sensitivity induced by a chronic low-level RF-EMF exposure on the thermoregulatory behavioral response, we monitored the thermal preference of exposed young rats. A juvenile model was used in order to simulate the physiological and behavioral responses to environmental stress in children and teenagers. The French Agency for Food, Environmental and Occupational Health and Safety (*Agence Nationale de Sécurité Sanitaire de l’alimentation, de l’environnement et du travail*) has recently recommended reducing the RF-EMF exposure (i.e. lowering the SAR) for this vulnerable population (n°2011-SA-0150, October 2013). Our experiments were performed in an environmental chamber in which the rat was free to choose between three T_a_ values (24°C, 28°C and 31°C, corresponding to the lower boundary, midpoint and upper boundary of the rat’s thermoneutral zone [11], [12]). Within this zone, homeothermia can be maintained by controlling changes in peripheral vasomotor tone and by modulating wakefulness (W) and sleep stage distribution (which in turn modulate metabolic heat production) [13], [14], [15]. The level of RF-EMF exposure was chosen to mimic the chronic exposure encountered in current life close to a mobile phone base station. As the emission of Global System for Mobile Communications (“GSM”) mobile phones shows a clear modulation one eighth of the time, i.e. 576 µs every 4.6 ms, the emission of a base station is smoother and more random; at least one of the antenna’s channels (the broadcast control channel) is emitting at any given time. The other emitting channels are activated in a pseudorandom manner (depending on the traffic) and cannot be mimicked by a fixed modulation structure. Hence, continuous emission provides a fair representation of the exposure near to a base station antenna. A maximum electric field intensity of around 1 V.m^−1^ is found at ground level 100–200 m in front of a base station antenna. Our previous study showed that this intensity of exposure does not induce central heating. In this work, this intensity was associated with a significantly lower tail skin temperature (1.21°C below the control value at a T_a_ of 31°C), independently of the sleep stage [4]. The present study aimed at assessing the changes in thermal preference and sleep stage distribution that may potentially be influenced by a difference in skin temperature [11], [16].

## Materials and Methods

### 2.1. Animals and Accommodation

The rats were treated in accordance with European guidelines on the care and use of laboratory animals. The study protocol was approved (permit number: 130412-07) by the nationally recognized Regional Directorate for Health, Animal Protection and the Environment (Amiens, France). At the end of the study, the animals were sacrificed by intraperitoneal overdose (200 mg.kg^−1^) of pentobarbital sodium (CEVA Santé Animale, Libourne, France).

Experiments were conducted on young (3-week-old) male Wistar rats (Centre d’Elevage René Janvier, Le Genest Saint-Isle, France) weighing 50–75 g at the start of the experiment. The two groups animals were studied in similar air-conditioned, sound-proofed climatic chambers (2.4 m×2.43 m×1.6 m) with strictly controlled air and wall temperatures (24±1°C), lighting (a 12 h/12 h light/dark cycle, lights on at 7 am and off at 7 pm, 200 lux), relative air humidity (40±5%), air velocity (<0.2 m/s, indicating convection-free conditions) and noise levels (<65 dB). The recording equipment was located in an adjacent room. Animals were housed in individual plastic cages (32 cm×35 cm×20.5 cm) within the chamber. Food (standard AO3 chow: 51.7% carbohydrate, 5.1% lipid and 21.4% protein, expressed as percentages of total energy content; SAFE, Augy, France) and tap water were available *ad libitum.* The animals were divided into two groups of nine: the rats in one chamber were exposed to RF-EMF (from their arrival in the laboratory onwards) and the rats in the other chamber served as non-exposed controls. Two series of experiments were performed, with 4 animals in one group and 5 in the other. The two groups of animals were allowed to adapt to the laboratory conditions for three weeks prior to surgery (for implantation of a subcutaneous telemetric sensor). The animals in the exposed group were also exposed to RF-EMF during this period.

### 2.2. Exposure to RF-EMF

The climatic chambers housing the exposed group were equipped with RF-EMF antennas fed by a generator. A radio-frequency power source (model RFS 900–64, RFPA, Artigues-près-Bordeaux, France) emitting a continuous-wave 900 MHz electromagnetic field was connected to a four-output divider, which simultaneously supplied 4 antennas (model 800–10465, KATHREIN-Werke KG, Rosenheim, Germany). Each antenna was a broadband, directional, vertically polarized gain antenna designed for indoor radio installations and measured 23 cm×14 cm×5 cm. The antenna’s operating frequency bands were 806–960 MHz and 1710–2700 MHz. The antennas were located horizontally in the climatic chamber, 80 cm above the exposed rats’ boxes and the environmental chamber used to determine the preferred temperature. This RF-EMF was modelled (in the absence of grids and trays) using a near-field transformation [17], in order to obtain a field intensity of 1 V.m^−1^. The inter-antenna distance (48 cm) was chosen in order to obtain an exposure that was as homogeneous as possible for each rat. Since we were not able to perform numeric computation of the SAR with a finite-difference time-domain method, the whole-body SAR (defined as the RF power absorbed per unit of tissue mass) was calculated as recommended [18]. The SAR was estimated to be 0.3 mW.kg^−1^ for rats aged 3 weeks and 0.1 mW.kg^−1^ for rats aged 8 weeks (i.e. at the start of the recording period).

The level of RF-EMF exposure was checked with a frequency-selective radiofrequency dosimeter (EME-SPY 121, Satimo, Plouzané, France) and a broadband radiation monitor (EMR-200, Narda-STS, Pfullingen, Germany) for each box position in both the exposed and the sham climatic chambers. Frequency-selective fields were measured in each climatic chamber with a log-periodic wide-band antenna (20 MHz–3 GHz, HE 200, Rohde & Schwarz, Meudon-la-Forêt, France) and a spectrum analyzer (9 kHz–3 GHz, FSH3, Rohde & Schwarz). The system’s input and reflected powers were measured with a dual-channel power meter (model NRVD, with NRVZ4 and NRVZ5 probes, all from Rohde & Schwarz). Measurements were performed *in situ*, with the grids at the bottom of the cages, the trays under the cages and the dosimeter was placed on a grid. The target level of 1 V.m^−1^ was achieved at ±3 dB, suggesting that the RF-EMF was only weakly distorted by the metal parts at the bottom of the cages. In order to assess potential changes in exposure over time, the RF dosimeter recorded a value every 4 seconds for 6 hours. The mean values measured were between 1.15±0.09 V.m^−1^ and 0.78±0.11 V.m^−1^.

The RF-EMF exposure was emitted for 23.5 hours per day, starting on the animals’ arrival in the facility and ending on the day of euthanasia. Emission was only interrupted for 30 minutes a day (from 6 pm to 6.30 pm), during animal care. In the climatic chamber housing the control rats, four white, similarly-shaped but empty boxes were used to mimic the antennas.

### 2.3. Surgery

After three weeks of exposure, nine rats were implanted with a subcutaneous telemetric sensor connected to a wireless transmitter (model TL11M2-F20-EET, Data Sciences International, St. Paul, MN, USA), weighing 3.9 g and measuring 33 mm×33 mm×14 mm in size) for electroencephalography (EEG) and electromyography (EMG). The telemetric implant had two pairs of leads (and two channels) for EEG and EMG electrodes extending out of the device’s body. The medical-grade stainless steel leads were covered with silicon tubing. For surgery, each animal was given an intraperitoneal injection of a mixture of ketamine (85 mg/kg, Virbac, Carros, France) and xylazine (13 mg/kg, Bayer HealthCare, Kiel, Germany). All efforts were made to minimize suffering.

Two miniature gold-plated screws (diameter: 1.08 mm; length: 7.8 mm, Surtex Screw Post Dores RS1-S1, Henry Schein, Alfortville, France) were implanted in the skull of the animal (above the dura mater), in order to record EEG activity. The telemetric implant’s electrodes were wound around the screws and anchored to the skull with acrylic dental cement (Dentalon, Henri Schein, Alfortville, France). In order to avoid any possible effect of the RF-EMF via the metal electrodes, the latter did not penetrate into brain tissue. Electromyography was used to discriminate between W and paradoxical sleep (PS). On each side of the body, the EMG electrode lead wires were placed in direct contact with the dorsal muscles of the neck (along the same bundle of muscle fibres).

As previous study, spectral analysis confirmed that there were no effects of RF-EMF exposure on the EEG signal measured at the electrodes [4].

### 2.4. The Preferred Temperature and Sleep

#### 2.4.1. Environmental chamber

Thermal preference was assessed using an environmental chamber placed in the climatic chamber. For the exposed group, the environmental chamber was placed under two RF-EMF antennas (with one antenna between the two compartments). For the control group, the environmental chamber was placed under sham antennas.

The environmental chambers were built in house on the basis of an existing design [9]. Each chamber had three interconnected compartments (50×40×40 cm) with acrylic walls (thickness: 6 mm) and doors (10×10 cm) between them. Food and water were available *ad libitum* in each compartment. T_a_ could be independently set and maintained in each of the three compartments, since the latter were thermally insulated by a 5 mm air gap. The T_a_ in each compartment was regulated by an individual heating box comprising a fan, a resistor (20 W, 220 volts), a proportional-integral-derivative regulator (CAL 3200), a K-type thermocouple and a digital display (all from Radiospares, Beauvais, France). To check the spatial uniformity of the temperature in each compartment, T_a_ was measured at six points at the animal’s head height (i.e. 5 cm above the floor) and was found not to vary by more than 0.2°C.

#### 2.4.2. Infrared imaging

In the rat, changes in the peripheral blood flow at the tail (which acts as a heat exchanger) can be measured easily. In our earlier study, the tail skin temperature was recorded with temperature probe located at the base of the tail [4]. The skin temperature will depend on where on the tail it is measured. To confirm previous data, the tail surface temperature was continuously recorded by thermography with an infrared camera (Optris PI, Messtechnik Schaffhausen GmbH, Berlin, Germany) located 50 cm above the animal in the environmental chamber’s middle compartment. This camera can accurately and rapidly measure the tail skin temperature on the basis of the infrared radiation emitted from the skin surface (wavelength: 8–12 µm). The camera has a precision of 2%, a sensitivity of 0.08°C (at a T_a_ of 23±5°C) and a spatial resolution of 160×120 pixels. This method provides a temperature map of the surface of the rat’s tail and the animal is not affected by the stress that would be induced by direct attachment of a temperature probe. Skin temperatures can thus be measured with a high degree of accuracy over the whole surface of the tail. Infrared data were acquired and digitized with a microcomputer and the skin temperature map was generated with image analysis software (Optris PI, Messtechnik Schaffhausen GmbH, Berlin, Germany). The skin emissivity of the rat’s tail was set to 0.97.

#### 2.4.3. Activity monitoring

Locomotor activity was measured with infrared sensors (Coulbourn Instruments, Allentown, PA, USA) located at the base of the back wall of each compartment in the environmental chamber. The sensor signals were collected by an infrared analyzer activity (Coulbourn Instruments) which transmitted the information to an analog card (L18–16S/C, Coulbourn Instruments). The software determined the type and duration of body movement made by the animal: no movement or small or large movements lasting less than 0.5 sec, between 0.5 sec and 2 sec and more than 2 sec. Locomotor activity was recorded every 60 sec.

#### 2.4.4. Sleep recording

Rats are nocturnal animals and thus sleep more during the light period of the circadian cycle. Polysomnographic data were recorded with a TL11M2-F20-EET telemetric transmitter (Data Sciences International) and three receivers (RPC-1, Data Sciences International), which were located under each compartment of the environmental chamber. The receivers were coupled together and connected to a matrix (Data Exchange Matrix, Data Sciences International), which in turn was connected to a computer located outside the climatic chamber. The data were recorded with the Dataquest A.R.T. software (version 4.2 Bronze, Data Sciences International). The EEG and EMG signals were amplified, digitized at a sampling rate of 128 Hz and then filtered (with pass bands of 0.3–30 Hz for EEG signals and 1–100 Hz for EMG signals).

#### 2.4.5. Protocol

At the start of the experiment, the two groups of 3-week-old animals were housed in separate climatic chambers and had *ad libitum* access to food and water. One group was continuously exposed to RF-EMF (except during 30 minutes of care per day, as specified above) and the other group was not exposed.

For three weeks, the two groups were accustomed to the environmental chamber, with similar T_a_ (24°C) in the three compartments. Every other day, each animal was housed in the environmental chamber for 4 hours during the light period (i.e. at a time of day when the animal would sleep). Locomotor activity was recorded, in order to check whether the animal developed a spatial preference. We ensured that the animals entered each of the three compartments and did not always sleep in the same compartment. In the present study, no animal was excluded on the basis of these criteria. The surgical and post-surgical procedures were performed over the following two weeks.

At the beginning of the sixth week of RF-EMF exposure, each instrumented rat was tested in the environmental chamber. The animal could freely move and choose the compartment at 24°C, 28°C or 31°C as a function of its thermal preference. The relative air humidity (measured with a hygrometer involved in the environmental control of climatic chambers) was kept constant at between 40% and 50%. The air velocity (measured with a hot globe anemometer: Testo 490, Testo, Forbach, France; accuracy ±0.05 m.s^−1^) was always between 0.05 and 0.15 m.s^−1^ (indicating natural convection conditions). Locomotor activity and sleep were recorded for 24 hours on three non-consecutive days, during which T_a_ values of 24°C, 28°C or 31°C in the compartments were set at random.

#### 2.4.6. Analysis

Our analysis focused on comparisons between the group of rats exposed to the RF-EMF and the group of non-exposed rats.

To determine the rat’s preferred temperature (i.e. the compartment preferred by the rat), locomotor activity was scored for three days and the time spent in each compartment was calculated. T_a_ of each compartment was measured continuously. Entering a compartment for less than one minute was not qualified as a choice and this value was discarded in further analyses. The average time spent in each compartment was expressed as a percentage of the total duration of recording and was calculated for each animal during the 12 h light period and the 12 h dark period. The time spent in each compartment (in minutes) was also averaged over successive two-hour periods.

The polysomnographic data were visually scored every 10 seconds (using Dataquest A.R.T. software, Data Sciences International) for wakefulness (W: low-amplitude, high-frequency EEG, and high-amplitude EMG), slow wave sleep (SWS: high-amplitude, low-frequency EEG, and low-amplitude EMG) and paradoxical sleep (PS: low-amplitude, high-frequency EEG, and no EMG muscle tone). “Active wakefulness” (active W, which corresponds to exploring or eating) was scored but not assigned to a specific T_a_, since the animal can cross the different compartments. We only noted T_a_ for “quiet wakefulness” (quiet W); these are short episodes (<2 minutes) of W that occur within sleep stages and during which the animal does not move or eat. The amounts of active and quiet W were scored on the basis of locomotor activity (no movement = quiet W) and the video recordings from the infrared camera. Episodes of SWS were defined as periods of sleep longer than 20 s that were preceded or followed by W or PS [19]. Episodes of PS were defined as periods of sleep longer than 20 s that were preceded by episodes of SWS (of any duration) or W (duration below 30 s). Only episodes of W or sleep occupying more than 60% of the 10 s analysis window were considered. The total sleep time (TST, i.e. the sum of SWS and PS) and the total amount of active W, quiet W, SWS and PS were expressed as a percentage of the overall analysis time. Due to the time used for caring animals, the average over the total analysis time is not the exact average of the light-time and the dark-time values. The mean durations (in minutes) and frequencies (the number of episodes per hour) of active W, quiet W, SWS and PS episodes were calculated. For each episode of quiet W and sleep, the T_a_ was noted.

The infrared tail scans were color-coded with the camera’s software (Optris PI, Messtechnik Schaffhausen GmbH, Berlin, Germany), with each of the nine colors representing a known temperature. The sensitivity between adjacent colors was set to 1°C, giving an overall temperature range of 9°C. Lastly, the infrared images were analyzed with in-house software designed to calculate the skin temperature all along the rat’s tail.

#### 2.4.7. Statistical analysis

The data were expressed as the mean ± standard error of the mean (SEM) and analyzed using Statview software (version 5.0, SAS Institute Inc., Cary, NC, USA). In view of the small sample size, non-parametric tests were used. A Mann-Whitney test was used to assess differences between the RF-EMF exposed group (n = 9) and the control group (n = 9). A Friedman test was used to test for thermal preference (i.e. differences in the ambient temperature chosen by the animals). When *Q*-values were significant, a Wilcoxon test was applied to probe differences between light and dark periods and between pairs of T_a_ values. The threshold for statistical significance was set to p<0.05.

## Results

**Figure 1:**
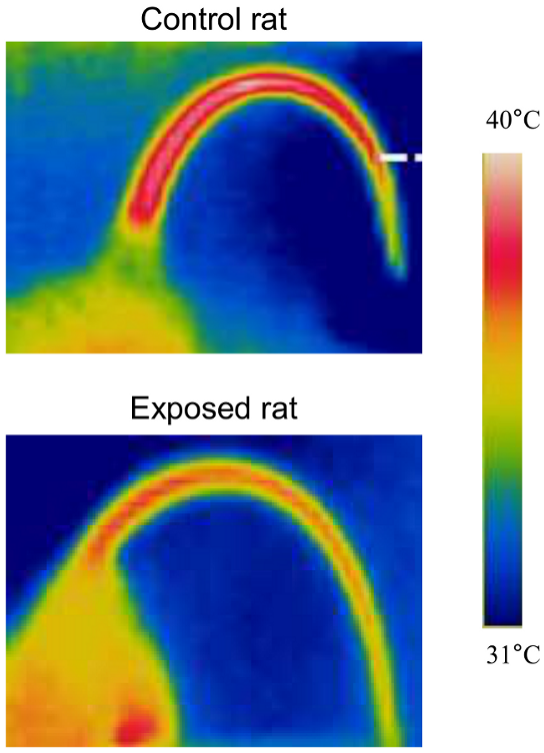
Thermal photographs of a control rat and an exposed rat at air temperature of 31°C. The colors correspond to the temperature scale on the right.

Figure 1 shows representative infrared thermographic images of a rat from the control group and a rat from the exposed group. When considering pooled data at a T_a_ of 31°C, the mean tail skin temperature was lower in the exposed group than in the control group (37.1±0.5°C and 38.7±0.4°C, respectively, i.e. a difference of 1.6°C; p = 0.049). An intergroup difference in tail skin temperature was not found at T_a_ values of 24 and 28°C.

During the adaptation period, all the rats met the starting criterion (i.e. a lack of preference between the three compartments). When comparing the three separate 24-hour recording periods for each rat, there were no significant differences in thermal preference or sleep/W parameters (p>0.32).

### 3.1. the Preferred Temperature

**Figure 2:**
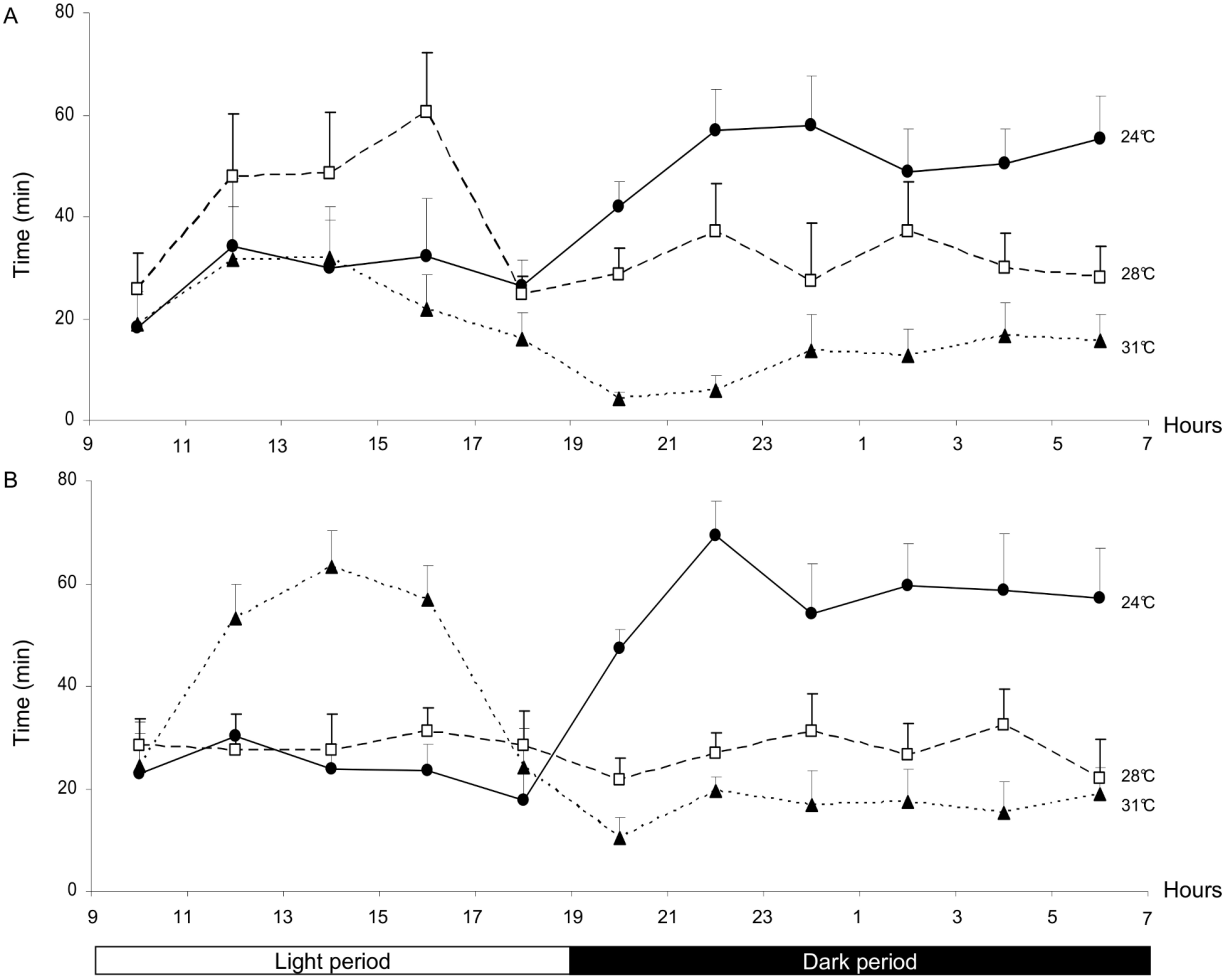
Thermal preference for controls (A) and RF-EMF exposed rats (B) over a 24 h period. The mean ± SEM time (in minutes, averaged over two-hour periods) spent in each of the three air temperatures zones (24°C (•), 28°C (□) and 31°C (▴), indicating the preferred temperature) during the dark and light periods.

We observed circadian variation in the animal’s thermal preference (Figure 2). During the light period, the thermal preference in the control group (top panel) was 28°C (with an acrophase between 3 pm and 5 pm). In the exposed group (bottom panel), the animals preferred 31°C during the light period (with an acrophase between 1 pm and 3 pm). The effect of RF-EMF exposure was significant during the light period only. During the dark period, the animals in both the control group and the exposed group preferred a T_a_ of 24°C, and the circadian variation in thermal preference tended to disappear (i.e. the curves reached a plateau) at 28°C and 31°C.

**Figure 3:**
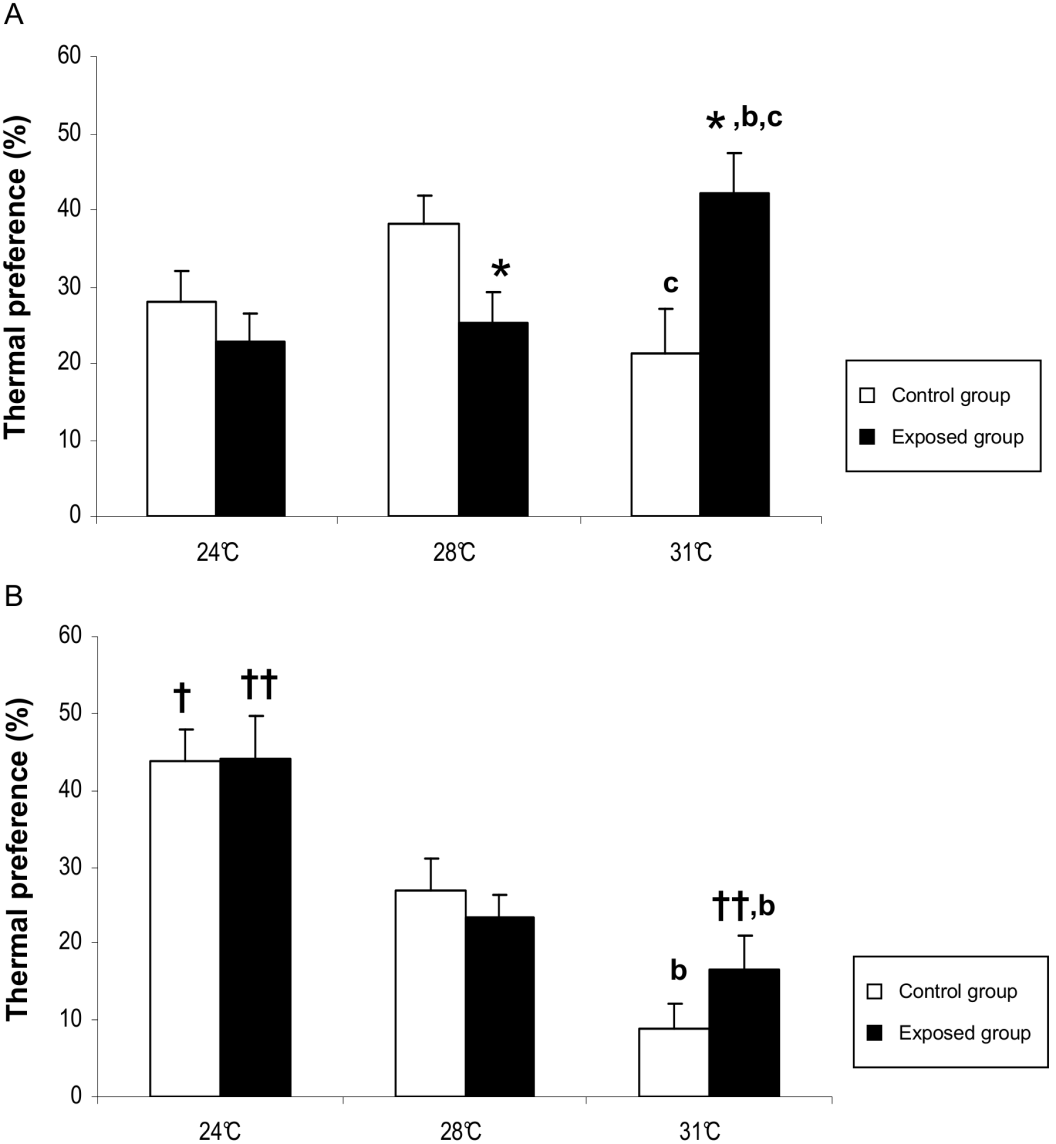
Thermal preference during the light period (A) and the dark period (B). The mean ± SEM time (as a percentage of all analysis time) of control rats (open columns) and RF-EMF-exposed rats (filled columns) for the three air temperatures values (24°C, 28°C and 31°C). Statistically significant differences are indicated as follows: (*) p<0.05, control group *vs.* exposed groups; (†) p<0.05 and (††) p<0.01 light period *vs.* dark period; (b) p<0.05, 24°C *vs.* 31°C; (c) p<0.05, 28°C *vs.* 31°C.

These observations were confirmed when we considered the mean values of the time spent in each of the three T_a_ zones (calculated separately for the dark and the light periods; Figure 3). In the light period, the control animals (open columns) spent more time at a T_a_ of 28°C than at 31°C (38.7±3.7% and 21.5±5.8%, respectively, p = 0.025). In contrast, the rats in the exposed group (filled columns) preferred a T_a_ of 31°C (42.5±4.8%) to a T_a_ of 24°C (21.8±4.0%, p = 0.049) or 28°C (25.9±4.2%, p = 0.049). This difference in thermal preference was confirmed when the animals in the exposed group were compared with those in the control group: +21.0% at 31°C (p = 0.015) and −12.8% at 28°C (p = 0.038). During the dark period, the thermal preference was 24°C and did not appear to depend on RF-EMF exposure. For example, both groups of animals spent more time at a T_a_ of 24°C than at 31°C (43.9±4.0% and 8.8±3.3%, respectively, p = 0.011 for the control group, and 43.9±5.7 and 16.6±4.2, respectively, p = 0.015 for the exposed group). For both groups of animals, the amount of time spent at a T_a_ of 24°C was greater during the light period than during the dark period (+15.7%, p = 0.049 for the controls and +24.2%, p = 0.008 for the exposed group). The animals in the exposed group spent less time at a T_a_ of 31°C during the dark period than during the light period (−28.1%, p = 0.008).

### 3.2. Sleep and the Preferred Temperature

Table 1 gives the TST and the various parameters related to W, SWS and PS for each of the two groups and for the analysis time as a whole (i.e. regardless of the chosen temperature zone). As expected in nocturnal rodents, the total sleep time (TST) was longer during the light period than during the dark period for both the control and exposed groups (+21.7% and +17.8%, respectively, p = 0.008), as a result of a greater total amount of SWS (+21.7%, p = 0.008, in the 2 groups). The light vs. dark difference in the TST was less marked in the exposed group, since the total amount of PS during the light period was lower than during the dark period (−4.0%, p = 0.008) and lower than in the control group (−2.7%, p = 0.047). In the control group, there was no significant difference in the total amount of PS between the light and the dark periods. In the exposed group, PS episodes were less frequent (−7.7 episodes.h^−1^, p = 0.01) and shorter (−0.8 min, p = 0.008) during the light period than during the dark period. In the control group, only the mean episode duration was shorter during the light period than during the dark period (−0.4 min, p = 0.008). The total amount of quiet W was longer during the light period in both groups (controls: +5.7%, exposed group: +5.1%, p = 0.008), since quiet W episodes were more frequent during the light period than during the dark period (controls: +10.7 episodes.h^−1^, p = 0.008; exposed group: +10.1 episodes.h^−1^, p = 0.008). The total amount of active W was greater during the dark period than the light period in both groups (controls: +26.8%, p = 0.012; exposed group: +23.3%, p = 0.008) as a result of the greater mean duration of W episodes (controls: +6.0 min, p = 0.015; exposed group: +7.5 min, p = 0.008). There was no significant difference in the frequencies of W episodes when comparing dark and light periods. When considering 24 h periods, only quiet W episodes were less frequent in the exposed group than in the control group (−4.5%, p = 0.015).

**Table 1:** Sleep structure parameters regardless of air temperature in the control and the RF-EMF exposed groups.

Stage	Parameters	Period	Control group	Exposed group
Active W	Total duration (%)	light	26.4±4.2	25.7±1.6
		dark	48.2±1.8^†^	49.0±3.3^††^
		24 h	37.5±1.0	38.2±2.0
	Frequency (episodes.h^−1^)	light	2.1±0.4	1.8±0.1
		dark	1.9±0.2	2.1±0.2
		24 h	2.0±0.2	2.0±0.2
	Mean episode duration (min)	light	10.1±0.8	8.4±0.7
		dark	16.1±1.5^†^	15.9±1.3^††^
		24 h	13.1±1.0	12.2±0.7
Quiet W	Total duration (%)	light	11.3±0.7	11.7±1.1
		dark	5.6±0.6^††^	6.6±0.6^††^
		24 h	7.9±0.5	9.0±0.7
	Frequency (episodes.h^−1^)	light	20.8±1.4	20.0±2.4
		dark	10.1±1.1^††^	9.9±1.2^††^
		24 h	16.7±1.0	12.2±1.2*
TST	Total duration (%)	light	62.9±4.3	62.3±2.7
		dark	41.2±2.5^††^	44.5±2.2^††^
		24 h	49.7±1.4	53.6±1.1
SWS	Total duration (%)	light	54.5±3.6	56.6±2.7
		dark	32.8±2.1^††^	34.9±1.9^††^
		24 h	41.3±1.4	45.0±1.6
	Frequency (episodes.h^−1^)	light	20.8±1.2	20.3±1.9
		dark	31.9±9.8	31.6±9.9
		24 h	15.1±0.8	15.4±1.1
	Mean episode duration (min)	light	1.4±0.1	1.6±0.1
		dark	1.6±0.1	1.8±0.2
		24 h	1.5±0.1	1.7±0.2
PS	Total duration (%)	light	8.4±1.0	5.7±0.7*
		dark	8.4±0.6	9.7±0.6^††^
		24 h	8.2±0.5	7.9±0.4
	Frequency (episodes.h^−1^)	light	3.9±0.4	3.1±0.3
		dark	8.2±2.2	10.8±3.7^†^
		24 h	3.4±0.2	3.4±0.2
	Mean episode duration (min)	light	1.0±0.1	0.8±0.1
		dark	1.4±0.1^††^	1.6±0.1^††^
		24 h	1.2±0.1	1.2±0.1

Mean ± SEM values for active wakefulness (active W), quiet wakefulness (quiet W), total sleep time (TST), slow wave sleep (SWS) and paradoxical sleep (PS) for the light and dark periods and during 24 h periods. Amounts are in fact expressed as a percentage of the analysis time, which due to animal caring, is less than 24 h. Statistically significant differences between light and dark periods are represented as follows: (^†^) p<0.05, (^††^) p<0.01. Differences between the control and exposed groups are indicated as follows: (*): p<0.05.

Table 2 gives the sleep structure parameters as a function of the chosen T_a_. The most striking results were found when the sleep data were analyzed for each T_a_ value. At a T_a_ of 31°C, the TST was greater in the exposed group than in the control group (+15.5%, p = 0.009) because of a greater total amount of SWS (+14.6% in the exposed group, p = 0.009). Quiet W was also greater in the exposed group than in the control group (+2.6%, p = 0.021). In turn, these relative increases in SWS and quiet W were due to greater episode frequencies (+4.9 episodes.h^−1^ for SWS in the exposed group, p = 0.021; +4.2 episodes.h^−1^ for quiet W in the exposed group, p = 0.034). There were no intergroup differences in the mean duration per episode of SWS or quiet W. When the animals in the exposed group chose a T_a_ of 28°C, the frequency of SWS episodes (−3.6 episodes.h^−1^, p = 0.038) and the total amount of PS (−2.1%, p = 0.027) were lower than in the control group. When considering 24 h periods at T_a_ = 31°C, the TST was greater in the exposed group than in the control group (+10.9%, p = 0.004) as a result of a greater amount of SWS (+6.9%, p = 0.038). Similarly, the total amount of quiet W was greater in the exposed group at a T_a_ of 31°C (+1.5%, p = 0.024). Conversely, at T_a_ = 28°C, the TST was lower in the exposed group than in the control group (−6.4%, p = 0.038). In the exposed group, the frequency of quiet W was also lower when the animals chose to stay at T_a_ = 24°C (−2.9 episodes.h^−1^, p = 0.047) and 28°C (−2.6 episodes.h^−1^, p = 0.024). Hence, the most marked differences between the exposed and control groups were observed for the light period and a T_a_ of 31°C.

**Table 2:** Sleep structure parameters when given a choice between three air temperatures values.

			Control group			Exposed group		
Stages	Parameters	Period	24°C	28°C	31°C	24°C	28°C	31°C
Quiet W	Total duration (%)	light	3.7±1.0	5.0±0.7	2.6±0.7	3.1±0.7	3.4±0.6	5.2±0.9*
		dark	3.0±0.5	1.8±0.4	0.7±0.4	3.5±0.7	1.8±0.4	1.2±0.5
		24 h	3.2±0.5	3.2±0.4	1.5±0.4	3.4±0.6	2.6±0.4	3.0±0.4*
	Frequency (episodes.h^−1^)	light	6.9±2.0	9.2±1.5	4.7±1.3	5.6±1.4	5.4±0.9	8.9±1.8*
		dark	5.3±0.9	3.4±0.7	1.4±0.7	5.2±1.2	2.7±0.5	2.1±1.0
		24 h	7.0±0.8	6.2±0.8	3.5±0.6	4.1±1.0*	3.6±0.5*	4.5±1.1
SWS	Total duration (%)	light	19.8±5.6	22.6±2.8	12.1±4.0	14.7±2.6	15.2±2.0	26.7±4.1**
		dark	17.4±2.1	10.5±2.0	4.9±1.9	21.7±4.0	8.2±1.6	5.0±2.4
		24 h	17.5±1.8	15.6±1.8	8.1±2.4	18.4±3.0	11.6±1.6	15.0±2.3*
	Frequency (episodes.h^−1^)	light	7.0±1.9	9.2±1.3	4.5±1.4	5.3±1.2	5.6±0.8*	9.4±1.8*
		dark	5.9±0.6	3.4±0.6	1.9±0.8	2.9±0.6	2.9±0.6	1.8±0.8
		24 h	6.1±0.6	5.9±0.7	3.1±0.9	6.0±0.6	4.1±0.6	5.2±0.9
	Mean episode duration (min)	light	1.5±0.1	1.4±0.3	1.3±0.1	1.5±0.2	1.5±0.2	1.7±0.3
		dark	1.7±0.1	1.8±0.2	1.3±0.1	1.9±0.2	1.6±0.2	1.9±0.3
		24 h	1.6±0.1	1.6±0.1	1.3±0.2	1.7±0.2	1.6±0.2	1.8±0.3
PS	Total duration (%)	light	3.2±1.0	3.6±0.9	1.6±0.5	1.8±0.6	1.5±0.3*	2.4±0.3
		dark	4.4±0.5	2.8±0.6	1.1±0.5	5.3±0.8	2.6±0.6	1.7±0.8
		24 h	3.7±0.4	3.1±0.6	1.3±0.3	3.7±0.5	2.1±0.4	2.0±0.4
	Frequency (episodes.h^−1^)	light	1.3±0.4	1.8±0.4	0.8±0.2	0.9±0.2	0.9±0.2	1.3±0.2
		dark	1.7±0.2	1.0±0.2	0.5±0.2	2.0±0.3	1.0±0.2	0.6±0.3
		24 h	1.5±0.2	1.3±0.2	0.6±0.1	1.5±0.2	1.0±0.2	0.9±0.2
	Mean episode duration (min)	light	0.8±0.1	0.9±0.1	1.1±0.2	0.8±0.1	0.7±0.1	0.9±0.1
		dark	1.6±0.2	1.5±0.1	1.2±0.2	1.6±0.2	1.6±0.2	1.7±0.2
		24 h	1.2±0.1	1.2±0.1	1.2±0.2	1.2±0.1	1.2±0.1	1.3±0.1
TST	Total duration (%)	light	22.9±6.6	26.2±3.5	13.7±4.5	16.4±3.1	16.7±2.3	29.2±4.3**
		dark	21.9±2.6	13.3±2.5	6.0±2.3	27.0±4.7	10.8±2.1	6.7±3.2
		24 h	21.4±2.0	19.4±2.2	7.7±2.0	22.0±3.6	13.0±1.9*	18.6±2.5**

Mean ± SEM values for the different sleep stages in the control and the RF-EMF-exposed groups when given a choice between 24°C, 28°C and 31°C during light and dark periods and during 24 h periods. Amounts are in fact expressed as a percentage of the analysis time, which due to animal caring, is less than 24 h. Statistically significant differences between the control and exposed groups are indicated as follows: (*): p<0.05 and (**): p<0.01.

## Discussion

The present study sought to assess the thermoregulatory behavioral response of rats during chronic exposure to low-intensity RF-EMF. Analysis of behavioral responses is particularly relevant, since the latter can be implemented rapidly and enable an animal to avoid or escape from external environmental constraints once detected. Most previous studies have used operant selection methods, in which animals were trained to press a bar or pull a rope to select a T_a_ [5], [20]. These data were informative because the extensive training may have restricted the animal’s choice. In the present study, the parameters related to thermal preference are highly sensitive because the free-moving animals can easily choose the optimal environment through natural movement. In earlier studies of exposure to high-intensity RF-EMFs, the assessment of behavioral responses could have been biased by directly induced hypothalamic heating [5], [6]. In the present study, the RF-EMF parameters were chosen to simulate chronic low-intensity exposure in growing animals and without a direct increase in brain temperature. Thus, the behavioral thermoregulatory responses observed during a constraint-free procedure are only driven by peripheral phenomena associated with low-intensity RF-EMF exposure.

The tail skin temperature was lower in the RF-EMF-exposed group than in the control group. The magnitude of the difference in tail temperature recorded by infrared thermography was similar to that found in a previous experiment [4] with subcutaneous spot measurements (1.21°C). We had used a pharmacological approach to demonstrate that the lower skin temperature was due to a greater peripheral vasoconstrictor tone [4]. In the present study, we showed that the difference in tail skin temperature between the exposed and control groups was not dependent on the measurement site.

During the dark period, the animals select a thermally neutral environment in which homeothermia can be maintained primarily by increasing metabolic heat production (through increased nocturnal motor activities and food consumption) at a T_a_ of 24°C. During the light period, the controls preferred to stay at a T_a_ of 28°C. This finding agrees with literature data on (i) non-exposed rats recorded using a similar technical approach [9], [11] and (ii) definition of the thermoneutral zone according to peripheral and core body temperatures in different species of rats [12]. In fact, a T_a_ of 28°C is an optimal environment for sleep and prevents a possible fall of body core temperature during paradoxical sleep. In the present study, the preference for a T_a_ of 31°C in the exposed group (relative to the control group and the selected T_a_ of 24°C or 28°C) was associated with a greater TST. Interestingly, there was an intergroup difference in thermal preference during the light period but not during the dark period. We hypothesize that T_a_ values of 24°C and 28°C are perceived to be less comfortable for sleep by RF-EMF exposed animals, which therefore choose a warmer environment. The difference in thermal preference was also associated with a lower tail skin temperature in the exposed group. This finding suggests that an exposed animal senses a cold thermal stimulus (probably elicited by skin thermoreceptors) that reinforces its vasoconstrictor tone and prompts the selection of a warmer T_a_. As reported [21], [22], the brain temperature is not involved in behavioral thermoregulatory responses. The cold sensation is induced by specific ion channels were expressed by small-diameter neurons in the trigeminal and dorsal root ganglia (transient receptor potential menthol in humans and in the rat [23], [24]). In our study, these channels might have been activated (in the absence of cold stimulus) by RF-EMF exposure. In fact, previous studies have shown that exposure to 2450 MHz RF-EMF in a modified 800 W microwave oven was sufficient to change protein conformation [25], [26]. However, we cannot rule out the possibility that our present results were due to the absence of warm receptor responses as a result of RF-EMF exposure.

During the light period, the shift in the preferred temperature towards higher T_a_ values was associated with a greater TST. This finding confirmed that the choice of a T_a_ of 31°C did not compromise homeothermia. The behavioral selection of a suitable environment may be useful for consolidating sleep and retaining body heat: at a T_a_ of 31°C, the lower air-skin temperature difference means that body heat losses and metabolic heat production during sleep are lower.

We concluded that the observed changes in sleep stage distribution were specifically due to RF-EMF exposure, since (i) these changes were not found in controls at a T_a_ of 31°C and (ii) the control and exposed groups did not differ in terms of total amount of paradoxical sleep (which is known to be particularly depressed by environmental thermal stress). However (as shown in Table 1), there was an inversion in the distribution of PS (regardless of air temperature), with less PS during the light period (relative to the control group) and more during the following dark period. This observation reinforces the hypothesis whereby exposed animals sense a cold stimulus (since PS is particularly sensitive to cold exposure). As shown in Table 2, the frequency of PS episodes at 31°C was greater (albeit not significantly) in the exposed group during both light (+47%) and dark (+59%) periods. We did not observe the significant fragmentation of this sleep stage previously reported [4], although the animals in the latter study were not allowed to choose their T_a_. One can rule out the possibility that this particular situation could be sensed by the animals as an additional source of stress. In the present study, the greater TST in the exposed group resulted from a greater total amount of SWS. These differences might enable appropriate responses to homeostatic challenges and a potential lack of SWS [27]. In contrast, exposed animals had a lower frequency of SWS episodes and a lower total amount of PS at a T_a_ of 28°C. In the present study, RF-EMF exposure may have induced greater peripheral vascular conductance and thus a cardiovascular challenge. It is clear that the lower skin temperature involves arousal thermoregulatory processes, which oppose the maintenance of SWS [16], [28]. Thus, thermoregulation would be more efficient if the frequency of quiet W episodes increases. The change in the amount of SWS (through a change in the episode frequency) can be considered as an adaptation that protects against environmental disturbances and maintains both this sleep stage and PS episodes, since functional changes in SWS underlie the onset of PS. It is not possible to draw firm conclusions as to the processes involved in the SWS fragmentation observed in exposed animals at a T_a_ of 31°C, since the literature studies have focused on PS rather than SWS.

There are several possible explanations for the greater amount of SWS in exposed rats at 31°C during the light period. Firstly, levels of alertness are increased by RF-EMF exposure and moderate warming [16], [29]. As reported [30], [31], anything that increases alertness or makes an animal more aware of the environment (such as pain, anxiety and emotion) may increase the amount of SWS. In the present study, this cannot be the case because (i) the control and exposed groups did not differ significantly in terms of the total amount of SWS at T_a_ values of 24°C or 28°C and (ii) there was no effect of T_a_ on SWS in the control group. One could hypothesize that the greater amount of SWS in exposed animals was due to the accumulation of a sleep debt during the dark period at 31°C. However, this possibility can also be ruled out, since there was no intergroup difference in the amount of either TST or SWS during either the light or the dark periods or when considering 24 h periods. Thus, the exposed group’s thermal selection of a warmer temperature during the light period may help the animals to reduce their cold feeling and thus fulfil their normal sleep requirements. Our present data suggest that the sleep stage distribution (and particularly the greater frequency of SWS episodes) can be influenced by peripheral temperature inputs. The impact of peripheral thermal inputs on (i) sleep stage distribution, (ii) signaling thermal stress, and (iii) increasing SWS (independently of the central thermoregulation state) has been emphasized [16]. Thermosensitive neurons activated by peripheral thermosensors have been identified in the median preoptic nucleus and ventrolateral preoptic area, both of which are involved in controlling the duration of sleep episodes [32], [33].

In conclusion, chronic exposure to RF-EMF in growing rats was associated with a shift in thermal preference towards a warmer T_a_ and a greater TST (due to a greater frequency of SWS). Our present results suggest that these changes are due to a change in the peripheral thermal sensation, which might result from modifications of specific peripheral thermoreceptors.

## Supporting Information

Arrive Guidelines S1 (PDF)

Arrive Guidelines S2 (PDF)
